# Present Status of Radio-Active Therapy

**Published:** 1914-08

**Authors:** 


**Present Status of Radio-Active Therapy.** Ernest Zueblin, Baltimore, Md. Med. Jour., June, 1914, extensive bibliography. (Note: Our exchange copy is on file at the Grosvenor Library, Buffalo).

